# Integrating climate change and fine-scale habitat suitability to assess amphibian range shift in Mount Emei, China

**DOI:** 10.1186/s12983-025-00570-6

**Published:** 2025-07-29

**Authors:** Zijian Sun, Tian Zhao, Suwen Hu, Guangfeng Chen, Wei Zhu, Xiwen Peng, Shengqi Su

**Affiliations:** 1 College of Fisheries, Southwest University, Chongqing, 400715 China; 2 CAS Key Laboratory of Mountain Ecological Restoration and Bioresource Utilization and Ecological Restoration Biodiversity Conservation Key Laboratory of Sichuan Province, Chengdu Institute of Biology, Chinese Academy of Sciences, Chengdu, 610041 China

**Keywords:** Ensemble models, Mountainous ecosystems, Climate change, Habitat suitability

## Abstract

**Background:**

Habitat range shifts driven by climate change threaten global biodiversity, with impacts likely to be most pronounced at mountainous regions. As key consumers, amphibians play critical roles in mountainous ecosystems by contributing to nutrient cycling, soil aeration through burrowing activities, and aquatic bioturbation. However, they are particularly vulnerable to climate change due to limited mobility and strong dependence on ambient temperature. Understanding their ranges shifts and responses to various environmental factors is a priority for identifying target conservation areas at a local scale. Here, we developed ensemble models to examine the current distribution of suitable habitats for amphibians, identify the environmental determinants of these habitats, and predict the potential range shifts under different climate projections in 2055 and 2085 in Mount Emei, China.

**Results:**

Our results indicated that lowland areas exhibited higher geographical habitat suitability for amphibians, which serve as a transitional zone between urban regions and forests. The current distribution of amphibians is primarily associated with the Normalized Difference Vegetation Index and climate variables related to precipitation and solar radiation. This pattern may be attributed to amphibians’ physiological constraints and their specific requirements for food and habitat. Interestingly, even in a region developed for tourism, anthropogenic factors exhibited a positive correlation with amphibian distribution. This may be explained by the high habitat suitability found in lowland suburban regions. Moreover, the small body size of amphibians allows them to thrive in smaller, specialized habitats. Looking toward the future, geographical habitat suitability for amphibians projected to decline, particularly in lowland suburban areas under the high-emission and high-carbon consumption scenarios. These areas currently represent important habitats for amphibians but are expected to experience substantial degradation. These findings highlight the need for targeted conservation efforts in areas currently providing high suitability for amphibians, which may face increased pressure over time.

**Conclusions:**

This study identifies the key determinants of amphibians’ current habitat suitability and illustrates a projected decline in overall habitat suitability under future climatic scenarios in Mount Emei. Future research on amphibian range shifts are encouraged to integrate considerations of their limited dispersal capacity and unique ecological characteristics.

**Supplementary Information:**

The online version contains supplementary material available at 10.1186/s12983-025-00570-6.

## Background

Contemporary climate change has emerged as a prominent and ongoing global phenomenon, increasingly recognized as a dominant driver of biodiversity change and potential species persistence challenges in the near future [1–3]. The global biodiversity crisis is exacerbated by climate change-induced alterations, which affect biodiversity at multiple levels [4–6]. A comprehensive understanding of species’ responses to climate change is essential for developing effective mitigation strategies to address this biodiversity crisis.

Shifts in the geographic distribution of species are a common consequence of climate change, with the potential to fundamentally restructure ecological communities and profoundly alter ecosystem structures and functions [7–9]. For instance, Zhu et al. [10] predicted that the climatically suitable breeding habitat ranges of Black-tailed Godwits (*Limosa bohaii*) may shrink by 86% by 2070, based solely on projected future climate, which could precipitate a potential decline in population size. Analogous research has extended beyond single taxonomic groups, revealing similar trends across multiple species and taxonomic categories (e.g., insects: [11, 12]; reptiles: [13, 14]; mammals: [15, 16]; birds: [17]; amphibian: [18, 19]). Given these patterns, it is imperative and urgent to understand how species distribution ranges will respond to future climatic change to implement effective, long-term conservation strategies.

As terrestrial ectotherms, amphibians are particularly vulnerable to climate change due to their limited mobility and pronounced dependence on ambient temperature [20, 21]. Luedtke et al. [22] highlighted that amphibians are the most endangered vertebrate class, with 40.7% of species globally threatened. The impacts of current and projected climate change contribute significantly to this growing crisis, accounting for 39% of the observed deterioration in amphibian conservation status since 2004 [22]. For instance, Adde et al. [23] demonstrated that amphibians are among the most affected groups in response to reductions in climate suitability under the Representative Concentration Pathway 4.5 (RCP 4.5: a mid-range emissions scenario) climate projections for Switzerland. For single species, such as *Andrias davidianus*, the world’s largest extant amphibian, climate-driven habitat alterations are projected to induce substantial range contractions and increased habitat fragmentation [24, 25]. However, despite such studies have been conducted, our understanding of how the distributions of multiple amphibian species to climate change at a local scale remains limited.

Despite occupying less than 20% of the Earth’s surface, mountainous regions host a rich diversity of terrestrial and aquatic habitats [26]. These areas serve as refugia for various species and could buffer the adverse impacts of climate change [27, 28]. Mount Emei, located in the transition zone between the Qinghai–Tibet Plateau and the Sichuan Basin in China (29°16′–29°43′N, 103°11′–103°37′E), is a key region where diverse natural elements converge [29]. Its unique geographical position and climatic conditions make it a complex terrain, with a wide elevational range (from 390 to 3090 m), providing an ideal research platform for studying the geographical distribution patterns of amphibians [30]. Amphibians play critical roles in mountainous ecosystems as consumers, influencing ecosystem structure and function through soil burrowing, aquatic bioturbation, decomposition, and nutrient cycling [31, 32]. Previous studies have reported on amphibian species composition [33, 34], spatial patterns of diversity [35, 36], and the mechanisms of community assembly [37, 38] along elevational and environmental gradients in mountains, highlighting the predominant influence of external environmental variables. However, a comprehensive understanding of amphibian range shifts due to climate change at a local scale is still lacking, despite their potential to significantly alter montane ecological communities [39, 40], presenting challenges for effective conservation efforts.

In the present study, we collected data of amphibian species distribution along with a set of current and future external environmental variables in Mount Emei. The aim was to indicate the determinants of amphibian spatial distribution and to assess potential changes in their habitat suitability in the near future. Specifically, we first examined the current suitable habitat for amphibians under multiple environmental stressors. We then identified key factors influencing amphibian distribution in Mount Emei. Finally, we predicted the potential habitat shifts under future climate projections and quantified the range shifts of amphibians in response to these climate scenarios. This research can provide valuable insights for the conservation and management of amphibians in Mount Emei, both under current conditions and in the face of future climate scenarios.

## Materials and methods

### Species occurrence records

We obtained amphibian occurrence records after 1970 primarily through historical specimen collections housed in the Herpetological Museum of Chengdu Institute of Biology (CIB), Chinese Academy of Sciences, as well as our recent field surveys. Additionally, we supplemented our database using resources from the Global Biodiversity Information Facility (GBIF, https://www.gbif.org/), AmphibiaChina (https://www.amphibiachina.org/), and relevant published literatures, based on the species list of Mount Emei (Additional file 1, Table S1). For records collected from GBIF, we retained only those labeled as “Human observation”, “Machine observation”, “Observation”, and “Occurrence”. To ensure data quality, we integrated all occurrence records and subsequently removed duplicates as well as records originating from institutes and museums using the “*dplyr*” and “*CoordinateCleaner*” packages, in R. After data cleaning, a total of 7978 occurrence records belonging to 35 species were compiled.

### Environmental variable for species distribution models

We selected four categories variables (i.e., climate, terrain, habitat suitability, and anthropogenic factors) as proxies for the main environmental processes and human disturbances influencing amphibian distribution [41]. Specifically, climate variability was represented by 19 bioclimatic variables (bio1-19), climate moisture index (CMI), near surface relative humidity (Hurs), mean solar radiation (MSR), and potential evapotranspiration (PET). Terrain variables included elevation (Ele), aspect (Asp), and slope (Slo). Habitat suitability was represented by the Normalized Difference Vegetation Index (NDVI), while anthropogenic pressures were quantified using human footprint (HFP) and people count (PC). The HFP variable accounts for eight human pressures cumulatively [42]. A complete overview and detailed information on all variables are provided in Table 1. All variables were spatially resampled and averaged to match the resolution of the geographic cells using ArcGIS 10.8.

**Table 1:** Environmental variables used to predict habitat suitability of amphibians in the present study

Categories	Environmental variable	Abbreviation	Unit	Timescale	Resource
Climate variables	Mean annual air temperature	Bio1	°C	1981–2010	https://chelsa-climate.org/
	Mean diurnal air temperature range	Bio2	°C	1981–2010	https://chelsa-climate.org/
	Isothermality’	Bio3	°C	1981–2010	https://chelsa-climate.org/
	Temperature seasonality	Bio4	°C /100	1981–2010	https://chelsa-climate.org/
	Mean daily maximum air temperature of the warmest month	Bio5	°C	1981–2010	https://chelsa-climate.org/
	Mean daily minimum air temperature of the coldest month	Bio6	°C	1981–2010	https://chelsa-climate.org/
	Annual range of air temperature	Bio7	°C	1981–2010	https://chelsa-climate.org/
	Mean daily mean air temperatures of the wettest quarter	Bio8	°C	1981–2010	https://chelsa-climate.org/
	Mean daily mean air temperatures of the driest quarter	Bio9	°C	1981–2010	https://chelsa-climate.org/
	Mean daily mean air temperatures of the warmest quarter	Bio10	°C	1981–2010	https://chelsa-climate.org/
	Mean daily mean air temperatures of the coldest quarter	Bio11	°C	1981–2010	https://chelsa-climate.org/
	Annual precipitation amount	Bio12	kg m^−2^ year^−1^	1981–2010	https://chelsa-climate.org/
	Precipitation amount of the wettest month	Bio13	kg m^−2^ month^−1^	1981–2010	https://chelsa-climate.org/
	Precipitation amount of the driest month	Bio14	kg m^−2^ month^−1^	1981–2010	https://chelsa-climate.org/
	Precipitation seasonality	Bio15	kg m^−2^	1981–2010	https://chelsa-climate.org/
	Mean monthly precipitation amount of the wettest quarter	Bio16	kg m^−2^ month^−1^	1981–2010	https://chelsa-climate.org/
	Mean monthly precipitation amount of the driest quarter	Bio17	kg m^−2^ month^−1^	1981–2010	https://chelsa-climate.org/
	Mean monthly precipitation amount of the warmest quarter	Bio18	kg m^−2^ month^−1^	1981–2010	https://chelsa-climate.org/
	Mean monthly precipitation amount of the coldest quarter	Bio19	kg m^−2^ month^−1^	1981–2010	https://chelsa-climate.org/
	Climate moisture index	CMI	kg m^−2^ month^−1^	1981–2010	https://chelsa-climate.org/
	Near-surface relative humidity	Hurs	%	1981–2010	https://chelsa-climate.org/
	Mean solar radiation	MSR	kJ m^−2^ day^−1^	1970–2000	https://worldclim.org/
	Monthly potential evapotranspiration	PET	kg m^−2^ month^−1^	1981–2010	https://chelsa-climate.org/
Terrain variables	Elevation	Ele	m	–	https://worldclim.org/
	Aspect	Asp	–	–	https://worldclim.org/
	Slope	Slo	°	-	https://worldclim.org/
Habitat suitability	The Normalized Difference Vegetation Index	NDVI	–	2000–2023	http://www.resdc.cn/
Anthropogenic variables	Human footprint	HFP	–	2000–2020	https://www.worldpop.org/
	People count	PC	Number of people	2000–2020	https://www.worldpop.org/

Under future climate projections, we obtained CHELSA v2.1 bioclimatic variable from The Coupled Model Intercomparison Project Phase 6 (CMIP6) in 2055 (2041–2070) and 2085 (2071–2100), with a resolution of 30 arc-second [43, 44]. Specifically, we considered four general circulation models (i.e., GFDL-ESM4, IPSL-CM6A-LR, MPI-ESM1-2-HR, and MRI-ESM2-0) and two emission scenarios: Shared Socioeconomic Pathways 1-Representative Concentration Pathway 2.6 (SSP126), representing a strong mitigation climate change scenario, and Shared Socioeconomic Pathways 5-Representative Concentration Pathway 8.5 (SSP585), representing a high-emissions and low climate-policy intervention scenario [45]. Moreover, we also incorporated terrain variables (i.e., Ele, Asp, and Slo) into species distribution models because of their temporal stability over decadal timescales. The future climate variables were spatially resampled and averaged to match the resolution of the geographic cells using ArcGIS 10.8.

### Ensemble species distribution modeling and prediction

Given the limited occurrence records in Mount Emei, the study area for species distribution modeling was expanded to include the surrounding regions of the Sichuan Basin (Additional file 1, Figure S1). This spatial expansion was primarily justified by the ecological homogeneity of habitats across the basin and the inherently limited dispersal abilities of amphibians [46]. To ensure model reliability, only species with more than 20 occurrence records in the extended region were used for further analyses (Additional file 1, Table S1, [47, 48]).

Ensemble species distribution models were constructed to examine the current and future potential distribution ranges of each species based on occurrence records and predictor variables. Compared to single algorithms, ensemble models are generally considered more robust, as they can reduce individual model uncertainties for different species and improve the overall reliability of projections [49]. Specifically, we employed ten modeling algorithms: Artificial Neural Network (ANN), Classification Tree Analysis (CTA), Flexible Discriminant Analysis (FDA), Generalized Additive Model (GAM), Generalized Boosting Model, or usually called Boosted Regression Trees (GBM), Generalized Linear Model (GLM), Multiple Adaptive Regression Splines (MARS), Maximum Entropy (MAXENT), Random Forest (RF), and Surface Range Envelop (SRE), using generalized pseudo-absences with the “eRandom” method [50]. A random sample of 70% of the initial data was used for model training, while the remaining 30% was used for evaluation, with the process repeated 10 times for each algorithm. Model performance was assessed using the area under the receiver operating characteristic curve (ROC) and true skill statistics (TSS). We retained models with a TSS value higher than 0.6 and applied the TSS method to weight the models for ensemble modeling, ultimately producing a species habitat suitability map at a 30 arc-second resolution [41].

### Geographical habitat suitability index and centroid migration

Based on the results from the ensemble models, habitat suitability for each species was classified into four distinct categories: high suitable, moderate suitable, low suitable, and unsuitable habitat (Additional file 1, Fig S2-S6). This classification was performed using the Jenks Natural Breaks Classification in ArcGIS 10.8 [12, 18], which is designed to minimize variance within classes and maximize variance betweem classes, resulting to a more structured and accurate representation of habitat suitability levels [51]. We then applied a Geographical Habitat Suitability Index (GHSI) to summarize the spatial distribution of suitable amphibian habatats in Mount Emei (adapted from Mi et.al; [52]). This index was an assemblages-based metric that intergrates habitat suitability maps of all species at a 30 arc-second resolution. The GHSI for highly suitable habitat was calculated as the percentage of high-suitability classification within each grid cell. Similarly, GHSI values for moderate suitable, low-suitability, and unsuitable habitats were quantified using the same approach, with each category represented by the percentage of corresponding habitat classifications within each grid cell. Finally, we calculated the changes in the proportion of each habitat suitability category under projected future climate scenarios relative to current conditions.

The stadard deviational ellipse (SDE) of GHSI was used to quantitatively describe the potential range shifts and distribution centroid migration in ArcGIS 10.8. The SDE method is commonly used to capture spatial orientation and distribution characteristics for spatial visualization [53]. We selected the centriod of the SDE to represent the distribution centroid of amphibians [53, 54].

### Explanatory variables for current geographical habitat suitability index

The assciations between the current GHSI and explanatory variables were examined through multiple liner regressions, and GBM. The explannatory variables are listed in Table 1. We first tested the pairwise correlations of environmental variables using Spearman’s rank correlation. Following variable selection protocols established in previous amphibian distribution and diversity studies [34, 41, 55], only one variable was included when two variables exhibited a strong correlation (|r|> 0.8; [10]). As a result, 13 variables were selected for further analyses, including bio2, bio4, bio7, bio12, bio15, Hurs, MSR, PET, Asp, Slo, NDVI, HFP, and PC (Additional file 1, Figure S7). All environmental variables were standardized to facilitate comparison of multiple regression coefficients. For GBM, variable importance was used to assess the role of explanatory variables in predicting geographical high suitability [41]. GBM measured variable importance using relative influence, which was based on the frequency with which a variable is selected for splitting. Each split was weighted by its squared improvement to the model, and the results were averaged across all trees [56]. To facilitate interpretation, the relative contributions of variables were normalized on a cumulative scale of 100%, with high values indicating stronger predictive associations with the response variables [57]. All calculations and statistical analyses were performed in R 4.2.1 [58].

## Results

### Current geographical habitat suitability

**Fig. 1:**
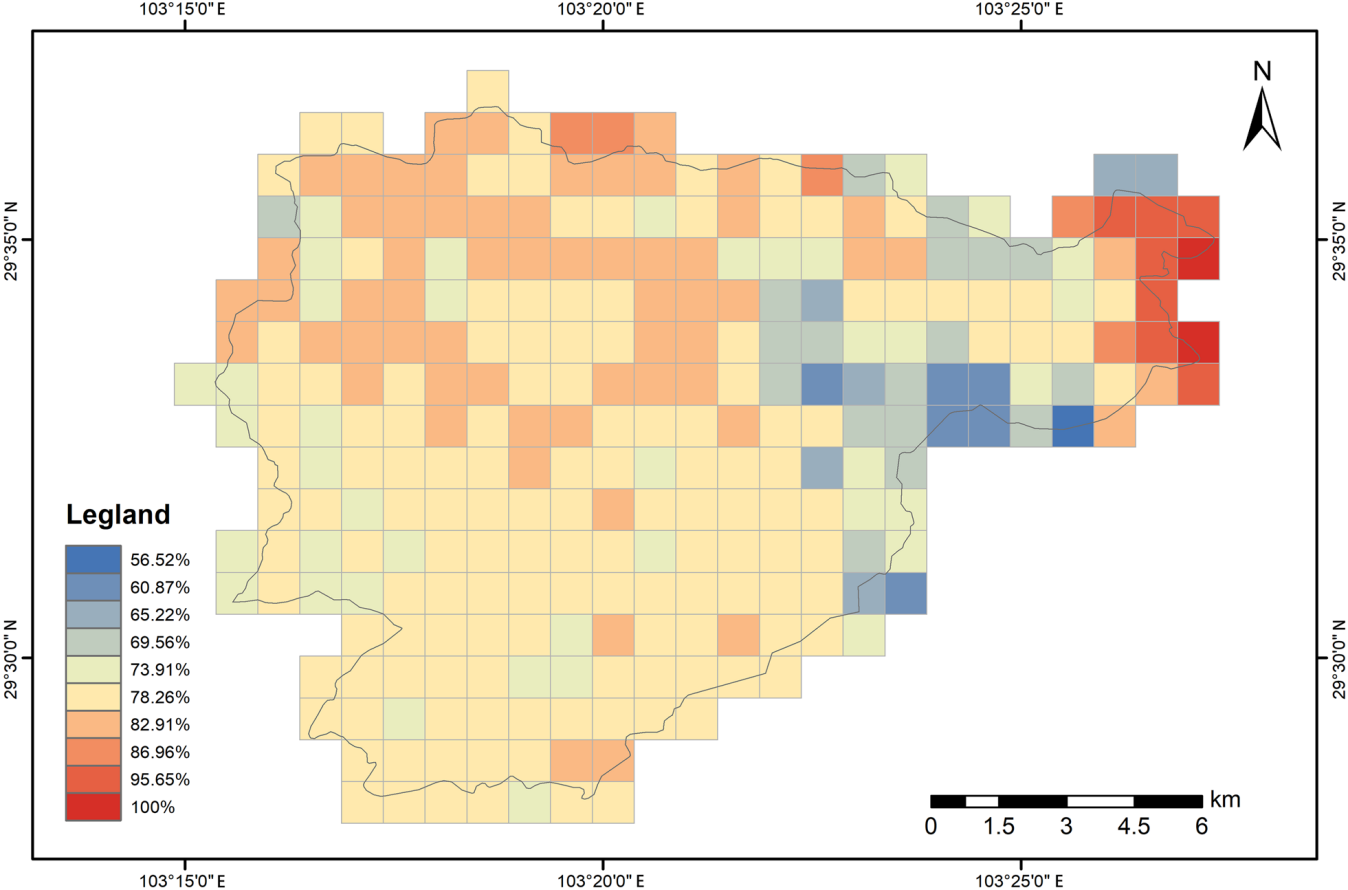
Current geographical habitat suitability index (GHSI) of high suitable habitats for amphibians in Emei Mount

According to the ROC and TSS values, the ensemble models for current species distributions performed well in this study (ROC: 0.941 ± 0.063; TSS: 0.993 ± 0.010; mean ± SD, and this format was used for all subsequent data presentations), demonstrating a satisfactory prediction with high reliability. The current geographical habitat suitability index revealed that highly suitable habitats predominantly occupied the grid cells, accounting for 56.52% to 100% of the area (Fig. 1). Moderate suitable habitats ranged from 0 to 26.09%, while low suitable and unsuitable habitats covered 0–17.39% and 1–8.70% of the areas, respectively. Due to the substantial proportion of highly suitable habitats, subsequent analyses of explanatory variables and SDE of the GHSI were focused specifically on high-suitability zones. Regions with higher values of geographical habitat suitability for amphibians were predominantly distributed in the lower elevation, peripheral areas along the northern edge and central sectors of Mount Emei (Fig. 1).

### Predominant predictors of current Geographical Habitat Suitability Index

**Fig. 2:**
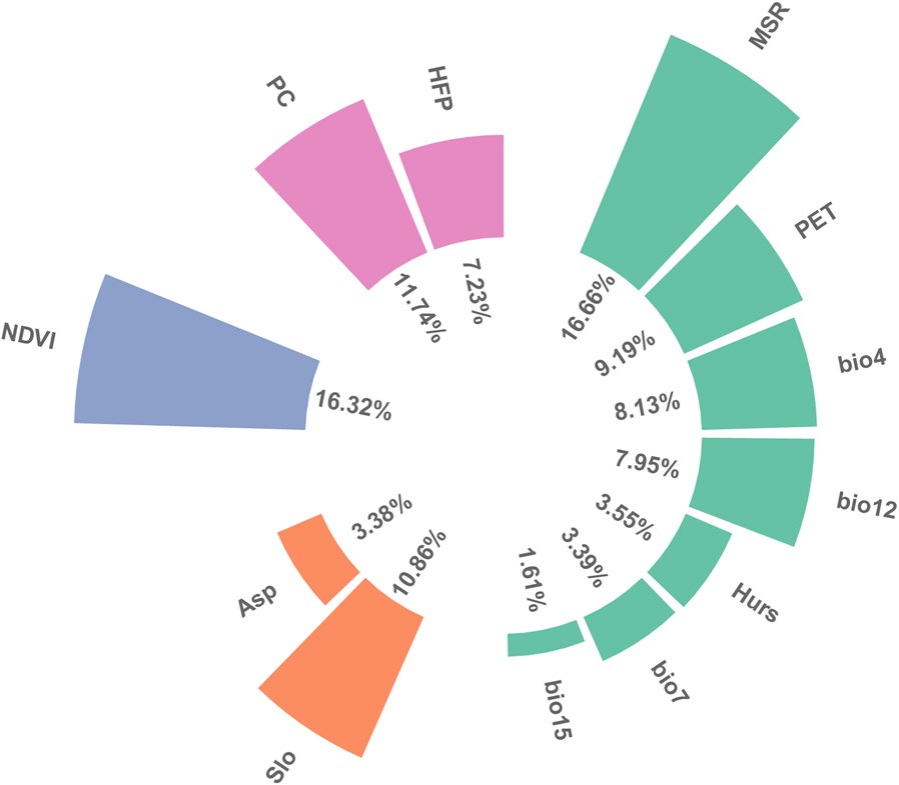
Contributions of environmental variables for current amphibian Geographical Habitat Suitability Index (GHSI) of high suitable habitats based on ensemble models. The abbreviation of each variable could be found in Table 1

The importance of explanatory variables for GHSI varied between the multiple liner regression and GBM model (Additional file 1, Table S2). The results identified MSR and NDVI as the variables most closely related to amphibian geographical habitat suitability in Mount Emei, with an importance value of 16.66% and 16.32%, respectively (Fig. 2). Climate variables, including PET, and bio12 also made significant contributions, with importance values of 9.19% and 7.95%, respectively (Fig. 2). Additionally, PC (11.74%), Slope (10.86%) and HFP (7.23%) played more subordinate roles in shaping the distribution patterns of amphibians in Mount Emei (Fig. 2).

### Potential change in geographical habitat suitability and centroid migration

**Fig. 3:**
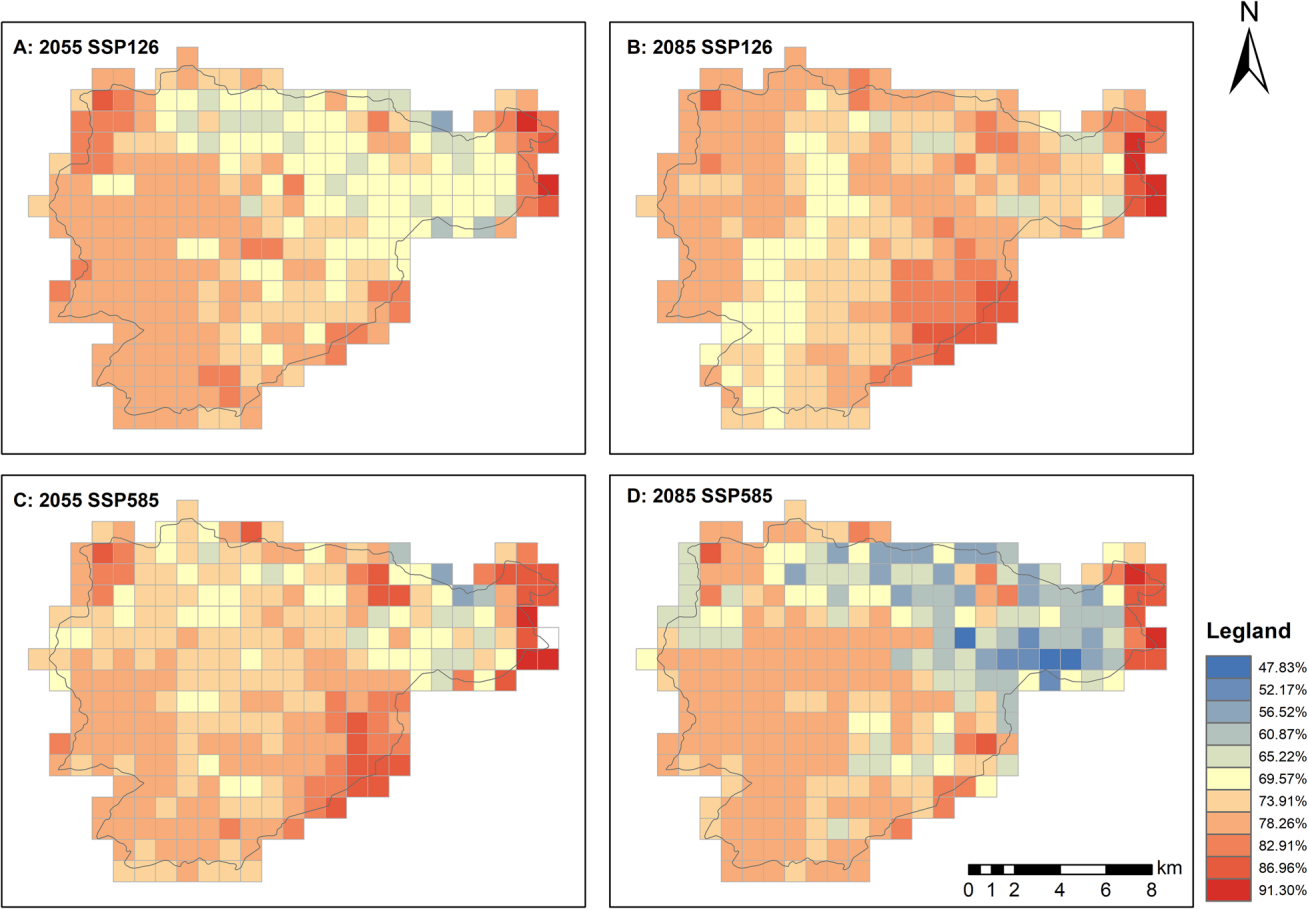
Geographical Habitat Suitability Index (GHSI) for amphibians in Mount Emei under future climate projections. A: In 2055 under SSP126 scenario; B: In 2085 under SSP126 scenario; C: In 2055 under SSP585 scenario; D: In 2085 under SSP585 scenario

According to the ROC and TSS values, the ensemble models performed well under projected future scenarios in the present study (ROC: 0.910 ± 0.071; TSS: 0.986 ± 0.014). The geographical habitat suitability for amphibians is expected to decline under both SSP126 and SSP585 scenarios by 2055 and 2085 (Table 2). Under the SSP126 scenario, 50.18% of high-habitat-suitability grid cells showed reduced suitability in 2055, with this percentage increasing to 53% by 2085 (Table 2, Fig. 3). In contrast, 49.82% of moderate-habitat-suitability grids exhibited suitability gains in 2055, which increased to 62.63% by 2085 (Table 2). For low suitable habitats, suitability declined in 67.16% of cells by 2055, with this proportion rising to 81.85% by 2085 (Table 2). Under the SSP 585 scenario, habitat suitability for high-suitability areas decreased in 49.11% of grid cells, with the affected proportion rising to 86.83% by 2085 (Table 2). In terms of moderate suitable habitats, 55.52% of grid cells showed improvements in habitat suitability, reaching 84.34% by 2085 (Table 2). Negative trends in habitat suitability were observed in 69.40% of low-suitability grid cells in 2055, which increased to 77.58% by 2085.

**Table 2:** The changes in each geographical habitat suitability category in 2055 and 2085 under different climate scenarios compared to the current suitable habitats

	2055 under SSP126 scenario	2085 under SSP126 scenario	2055 under SSP585 scenario	2085 under SSP585 scenario
High suitable habitat				
None	35.23	19.57	27.76	0.00
Down	50.18	53.74	49.11	86.83
Up	14.59	26.33	22.78	12.81
Moderate suitable habitat				
None	34.88	12.81	26.33	0.00
Down	15.30	23.84	17.44	14.95
Up	49.82	62.63	55.52	84.34
Low suitable habitat				
None	20.64	14.23	24.20	0.36
Down	67.26	81.85	69.40	77.58
Up	11.74	3.56	6.05	21.71
Unsuitable habitat				
None	25.27	20.64	29.89	8.54
Down	9.25	4.63	9.25	3.91
Up	64.77	74.02	60.14	86.63

None, done, and up indicated no changes, decline and improvement in geographical habitat suitability within the grid cells. Values indicated the proportion of grid cells with relative changes in geographical habitat suitability (%)

**Fig. 4:**
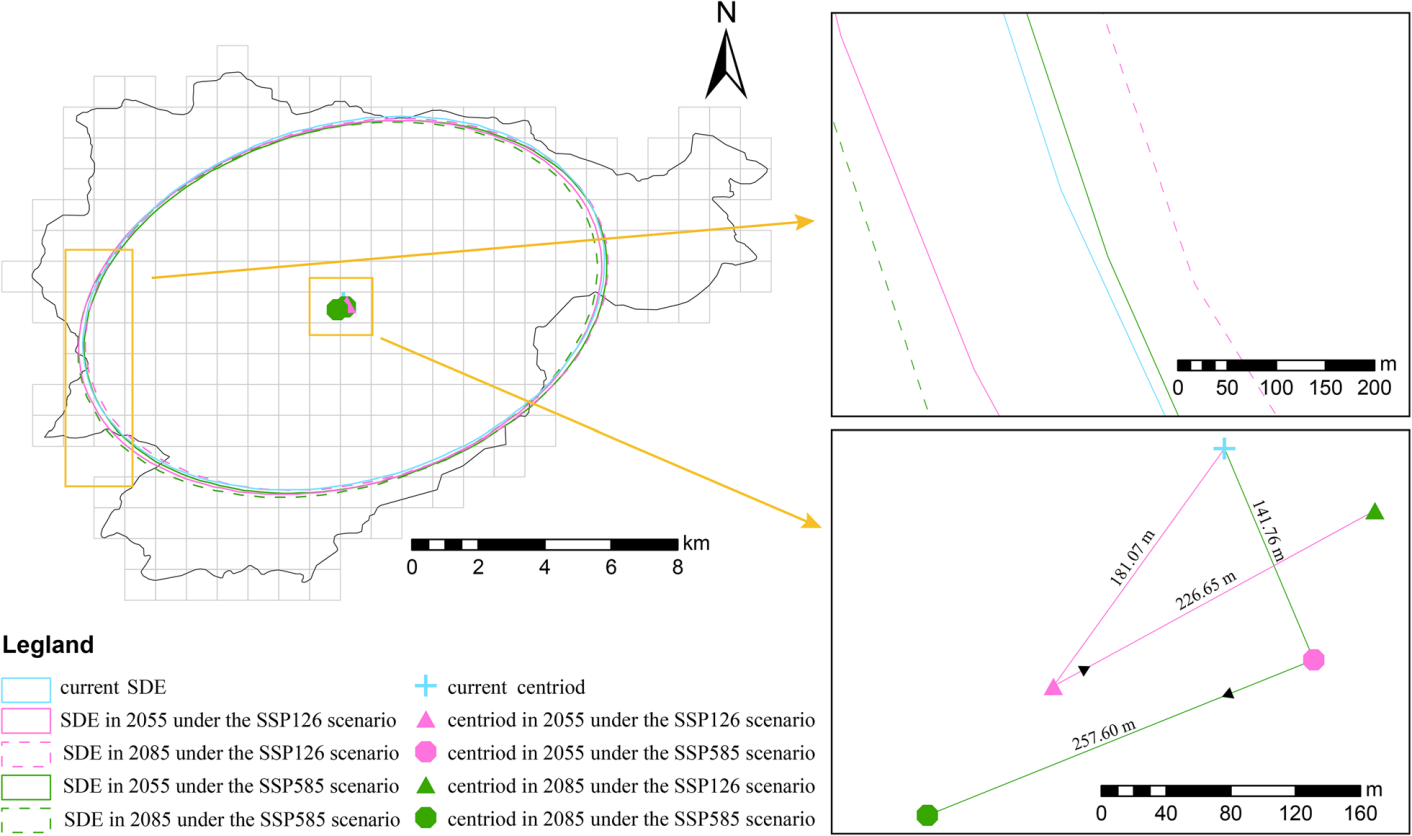
Standard deviational ellipse (SDE) of Geographical Habitat Suitability Index (GHSI) and its centroid migration under future climate projections in Mount Emei

In terms of centroid migration, under the SSP126 scenario, the centroid shifts to southwest by approximately 181.07 m by 2055, followed by a northeastward movement of 226.65 m by 2085 (Fig. 4, Additional file 1, Table S3). Under the SSP585 scenario, the centroid initially moves to southeast by141.76 m by 2055, then reverses direction, shifting southwest by 257.60 m by 2085 (Fig. 4, Additional file 1, Table S3).

## Discussion

In the present study, we used ensemble models to predict the determinants of current habitat suitability for amphibians and assess the potential habitat suitability changes in 2055 and 2085 under the SSP126 and SSP585 scenarios. Our results illustrated that, under current climatic conditions, habitats with high suitability for amphibians in Mount Emei are primarily distributed in the low and mid-elevation regions below 1500 m (e.g., the northeast and northwest areas), consistent with previous studies indicating high species richness in these regions [55]. These regions not only offer expansive areas of suitable habitats, but also serve as transitional zones between urban areas and forests. These regions feature diverse habitats (e.g., farmland, ponds, grasslands, and mountainous streams) that can support a wide range of species (e.g., *Microhyla fissipes*, *Fejervarya multistriata*, and *Pelophylax nigromaculatus*). Notably, the middle elevational areas (1850–2150 m) located in central Mount Emei were also identified as highly suitable habitats, particularly because they are the type locality for *Rhacophorus dugritei*, *Oreolalax major,* and *Rana omeimontis*, which requires cool temperatures and high humidity for breeding [59].

Our results indicated that NDVI was a prominent factor associating with the current suitability of amphibian habitats. High NDVI values typically indicate lush vegetation and high biomass, providing abundant food resources and suitable environments for amphibians [60]. Although lowland areas in Mount Emei exhibit lower NDVI values compared to pristine forests, they support greater habitat heterogeneity, which in turn sustains a higher diversity of species. This greater variety of habitats supports a wider range of amphibian species, enhancing overall habitat suitability in these regions. Additionally, Mi et al. [41] illustrated that mean annual precipitation had a significant effect on the spatial distribution patterns of amphibians, influencing their survival and reproduction at both species-specific and spatial levels. Our results corroborate this, highlighting the importance of precipitation-related climate variables such as potential evapotranspiration (PET) and annual precipitation in determining amphibian habitat suitability. These findings are also consistent with a prior study in Mount Emei, where PET and MSR played crucial roles in determining the composition of lowland amphibian communities [61]. Interestingly, despite the ongoing tourism development in Mount Emei, anthropogenic variables had a relatively lower, yet positive impact on amphibian distributions compared to other environmental variables like vegetation and climate. This could be attributed to the high geographical habitat suitability in the low-elevational suburban area. Moreover, our results confirm previous studies indicating that amphibians may exhibit greater resilience to human activities [41, 62, 63]. This resilience could be attributed to their small body size, which enables them to exploit specialized microhabitats and access food resources in a fine-scale environment [64].

The projected decrease in geographic habitat suitability for amphibians in Mount Emei under future climate scenarios contrasts with patterns observed in the Qinghai–Tibetan Plateau [65], where all species were combined into a single species distribution models. This approach treated all species as widespread generalists, neglecting the specific habitat requirements of specialists. The predicted decrease in habitat suitability suggests that many amphibian species, particularly those with narrow distribution ranges (e.g., *Scutiger chintingensis*), may face the challenge of seeking new habitats. Focusing on lowland suburban regions with currently high suitability for amphibians, the SSP126 low-emission scenario projects a strong decline in geographical habitat suitability by 2055. However, this decreasing trend is expected to stabilized by 2085, suggesting the potential for climate buffering through ecological adaptation or habitat shifting mechanism. In contrast, under the high-emission and high-carbon scenarios (SSP585), the area of highly suitable habitats for amphibians is projected to shrink, a trend that is anticipated to worsen over time. Such shifts may force species to move into new areas, but amphibians’ limited dispersal ability, especially in mountainous regions with physical barriers, presents a significant constraint on their potential for range expansion [66]. Our models assume unlimited dispersal, which might overestimate the real capacity of amphibians to colonize new areas as climate shifts. This assumption does not account for the significant barriers to movement that many species may face, making the future outlook for amphibians in Mount Emei possibly more precarious than suggested by the ensemble models. Given the high vulnerability of amphibians to climate change, as seen globally, the conservation of these species in Mount Emei may require urgent intervention to mitigate the risks posed by shrinking highly suitable habitats and limited dispersal capacity [67].

## Conclusion

In summary, the present study demonstrates that the current geographical habitat suitability of amphibians in Mount Emei are primarily determined by a combination of habitat suitability factors, climate variables, and anthropogenic pressures. Moving forward, it is recommended to continue exploring the effects of land-use change and other additional environmental stressors, while addressing potential biases in data reporting, sampling, and geographic scope. These efforts will help to enhance the accuracy and robustness of predictive models. The overall habitat suitability for amphibians is expected to decline, particularly under the high-emission and high-carbon scenarios. This underscores the importance of targeted conservation efforts in key regions of Mount Emei. This study also paves the way for further research on amphibian range shifts, emphasizing the need to incorporate species' limited dispersal capacity and ecological characteristics, such as behavior, physiological, and life history traits [68]. These factors are essential for understanding how amphibians will respond to climate change and for evaluating both interspecific and intraspecific interactions, which will ultimately shape their survival and adaptability in the face of environmental shifts.

## Supplementary Information

Additional file1

## Data Availability

The datasets used during the current study are available from the corresponding author on reasonable request.
