# Supplementary material for: Integrating climate change and fine-scale habitat suitability to assess amphibian range shift in Mount Emei, China
Source: Front Zool. 2025 Jul 29;22:16. doi: 10.1186/s12983-025-00570-6 (PMC12305991; doi:10.1186/s12983-025-00570-6)
Supplement: Supplementary file 1 — Additional file1 [file 12983_2025_570_MOESM1_ESM.docx]

**Integrating climate change and fine-scale habitat suitability to assess amphibian range shift in Mount Emei**

**Additional file 1**

**Table S1** The amphibian species list of Emei Mount. N_oc_ indicated the number of occurrence data in the range of buffer regions.

| **ID** | **Order** | **Family** | **Genera** | **Scientific name** | **N_oc_** |
| --- | --- | --- | --- | --- | --- |
| 1 | **Caudata** | **Cryptobranchidae** | *Andrias* | *Andrias davidianus* | 52 |
| 2 |  | **Hynobiidae** | *Batrachuperus* | *Batrachuperus londongensis* | 17 |
| 3 |  |  |  | *Batrachuperus pinchonii* | 49 |
| 4 | **Anura** | **Bufonidae** | *Bufo* | *Bufo gargarizans* | 314 |
| 5 |  | **Dicroglossidae** | *Fejervarya* | *Fejervarya multistriata* | 3 |
| 6 |  |  | *Nanorana* | *Quasipaa boulengeri* | 136 |
| 7 |  | **Hylidae** | *Hyla* | *Hyla annectans* | 59 |
| 8 |  | **Megophryidae** | *Atympanophrys* | *Atympanophrys shapingensis* | 12 |
| 9 |  |  | *Boulenophrys* | *Boulenophrys minor* | 28 |
| 10 |  |  |  | *Boulenophrys omeimontis* | 20 |
| 11 |  |  | *Leptobrachium* | *Leptobrachium boringii* | 27 |
| 12 |  |  | *Oreolalax* | *Oreolalax major* | 24 |
| 13 |  |  |  | *Oreolalax multipunctatus* | 5 |
| 14 |  |  |  | *Oreolalax omeimontis* | 18 |
| 15 |  |  |  | *Oreolalax popei* | 14 |
| 16 |  |  |  | *Oreolalax schmidti* | 12 |
| 17 |  |  | *Paramegophrys* | *Paramegophrys oshanensis* | 36 |
| 18 |  |  | *Scutiger* | *Scutiger chintingensis* | 10 |
| 19 |  | **Microhylidae** | *Kaloula* | *Kaloula rugifera* | 32 |
| 20 |  |  | *Microhyla* | *Microhyla fissipes* | 68 |
| 21 |  | **Ranidae** | *Amolops* | *Amolops chunganensis* | 33 |
| 22 |  |  |  | *Amolops granulosus* | 26 |
| 23 |  |  |  | *Amolops mantzorum* | 56 |
| 24 |  |  | *Hylarana* | *Hylarana guentheri* | 0 |
| 25 |  |  | *Nidirana* | *Nidirana daunchina* | 34 |
| 26 |  |  | *Odorrana* | *Odorrana graminea* | 13 |
| 27 |  |  |  | *Odorrana margaretae* | 113 |
| 28 |  |  |  | *Odorrana schmackeri* | 31 |
| 29 |  |  | *Pelophylax* | *Pelophylax nigromaculatus* | 109 |
| 30 |  |  | *Rana* | *Rana chevronta* | 4 |
| 31 |  |  |  | *Rana omeimontis* | 79 |
| 32 |  | **Rhacophoridae** | *Polypedates* | *Polypedates megacephalus* | 0 |
| 33 |  |  | *Zhangixalus* | *Zhangixalus chenfui* | 24 |
| 34 |  |  |  | *Zhangixalus dugritei* | 30 |
| 35 |  |  |  | *Zhangixalus omeimontis* | 52 |


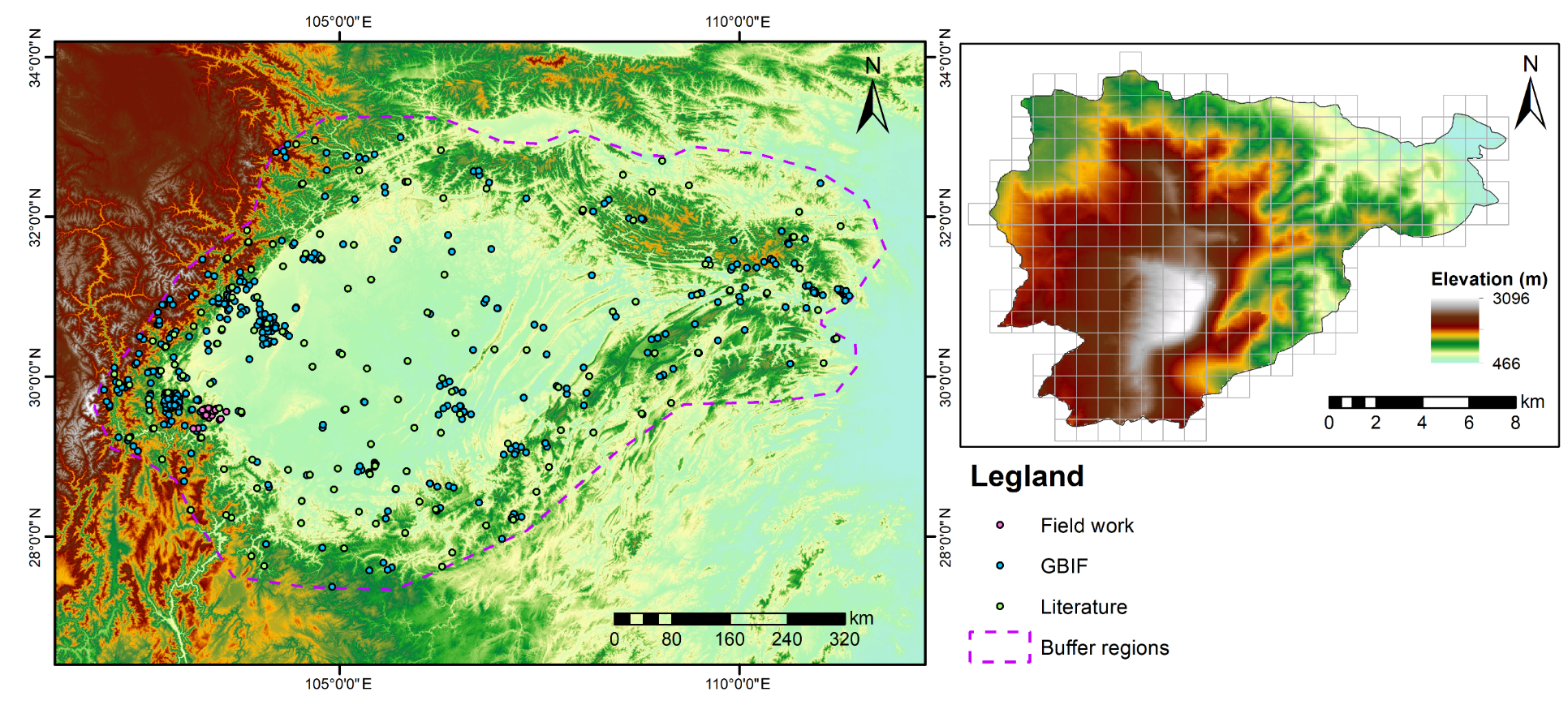


**Figure S1** Buffer regions and occurrence records used in the present study.


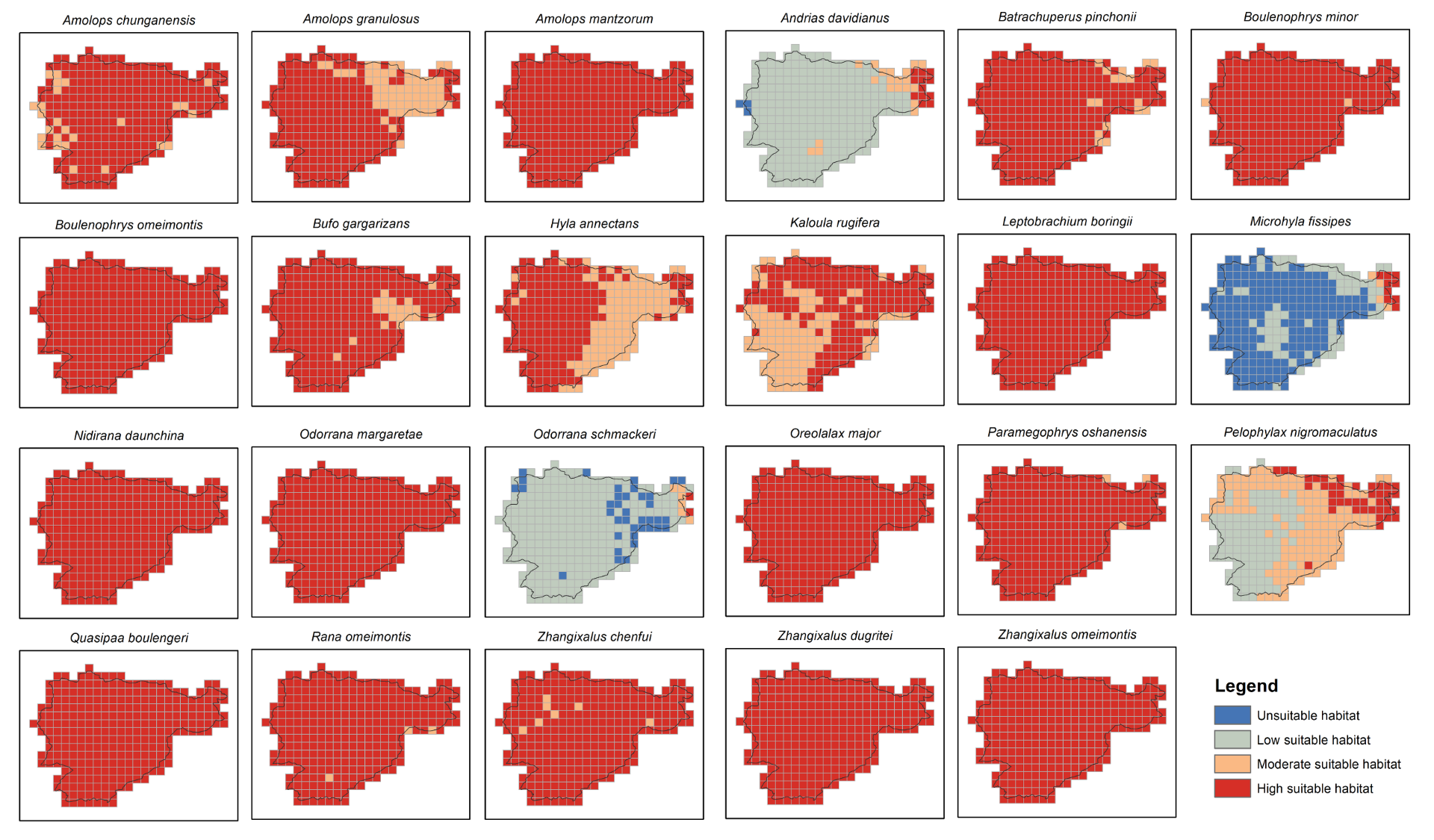


**Figure S2** Current suitable habitats for each selected species in the Mount Emei.


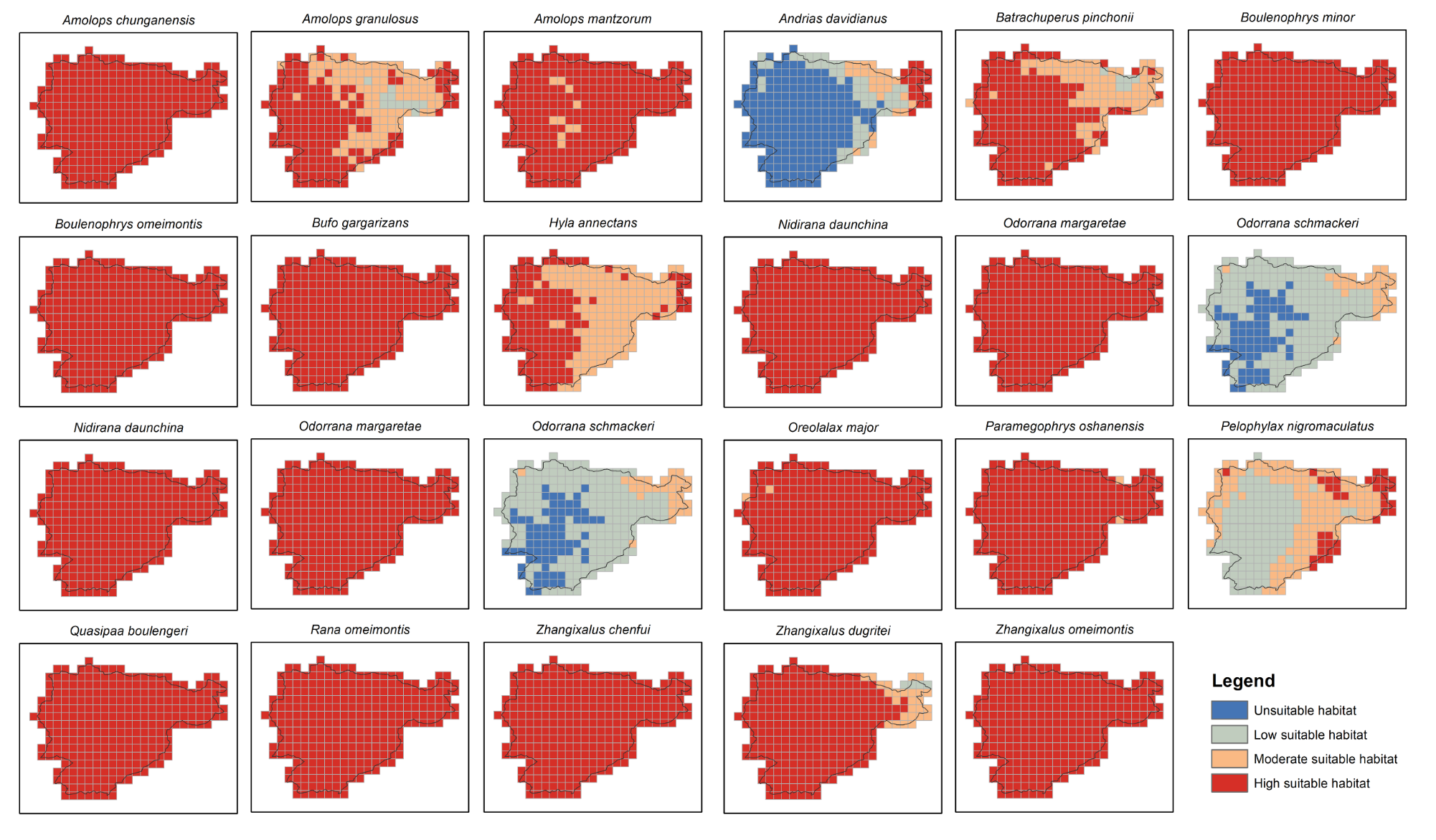


**Figure S3** Potential suitable habitats for each selected species in Mount Emei in 2055 under SSP126 scenario.


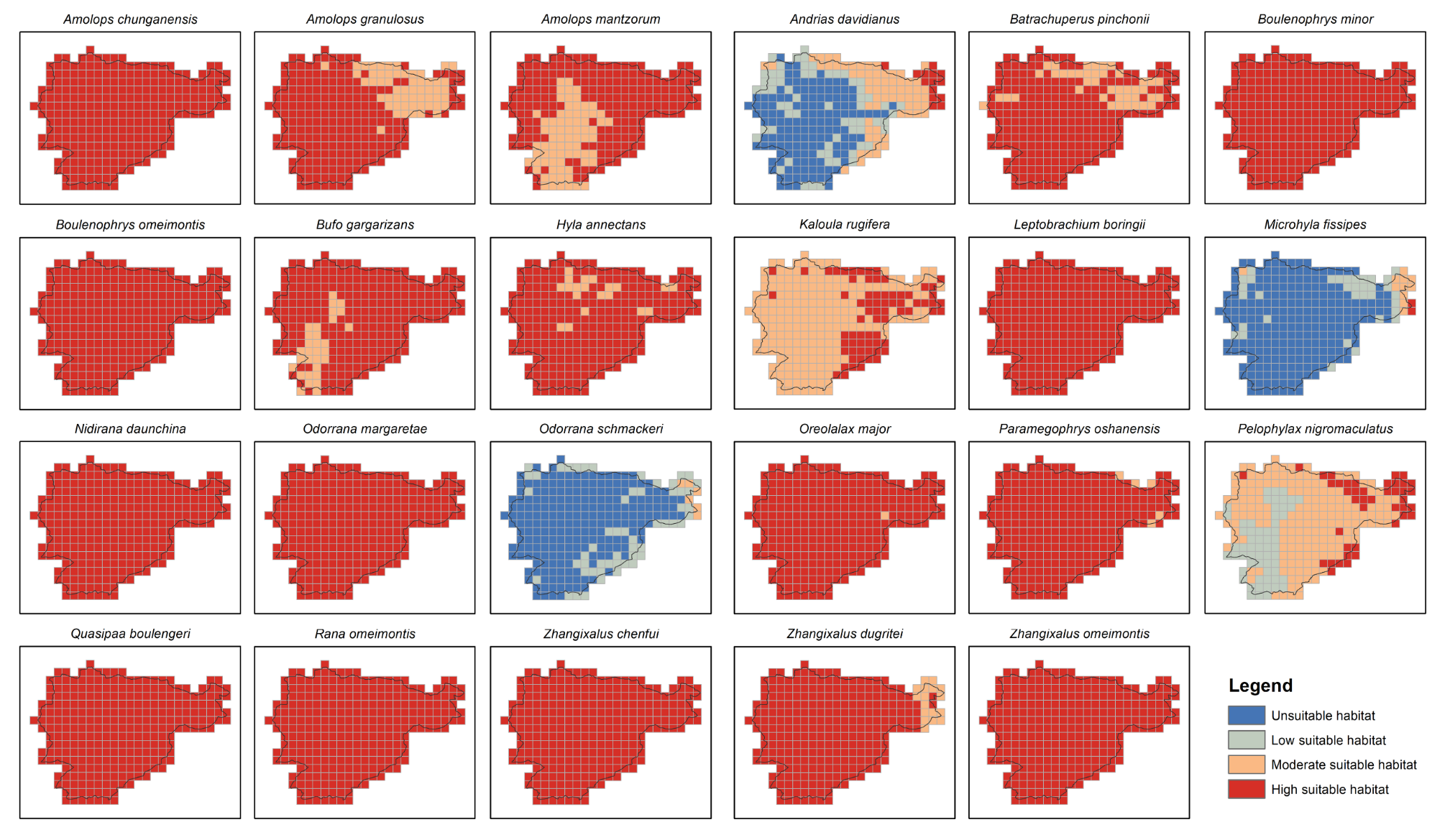


**Figure S4** Potential suitable habitats for each selected species in Mount Emei in 2085 under SSP126 scenario.


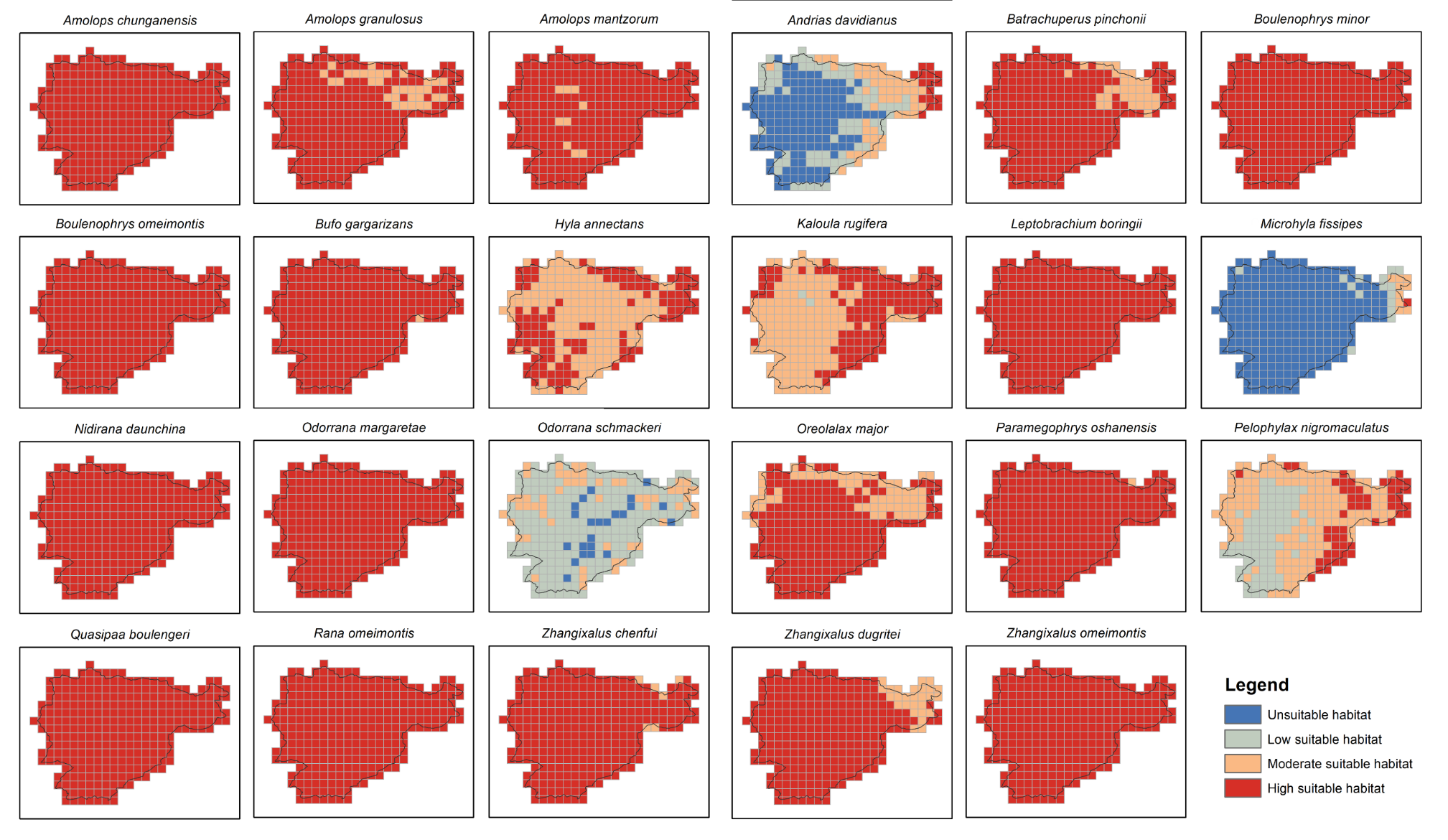


**Figure S5** Potential suitable habitats for each selected species in Mount Emei in 2055 under SSP585 scenario.


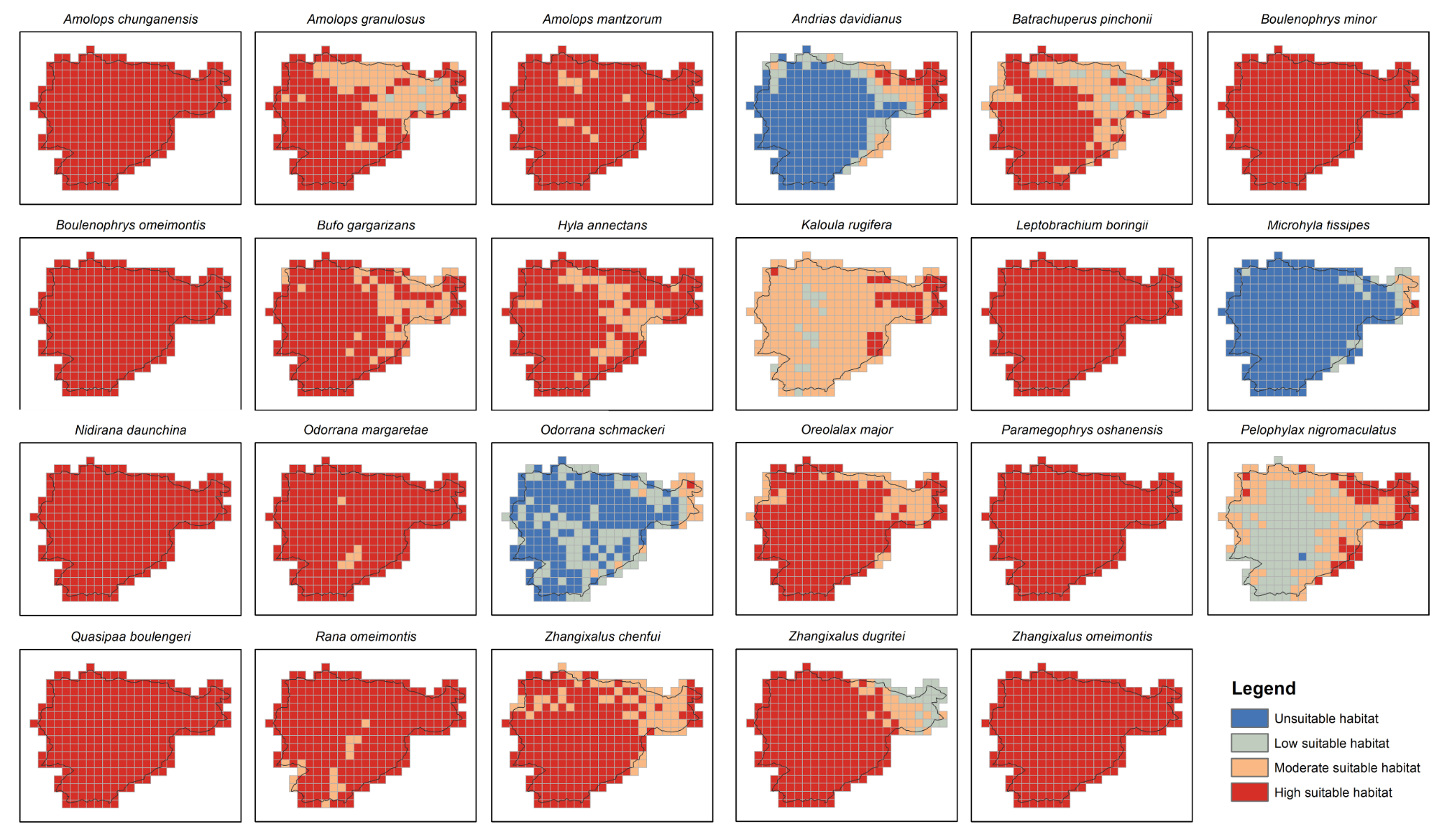


**Figure S6** Potential suitable habitats for each selected species in Mount Emei in 2085 under SSP585 scenario.

**
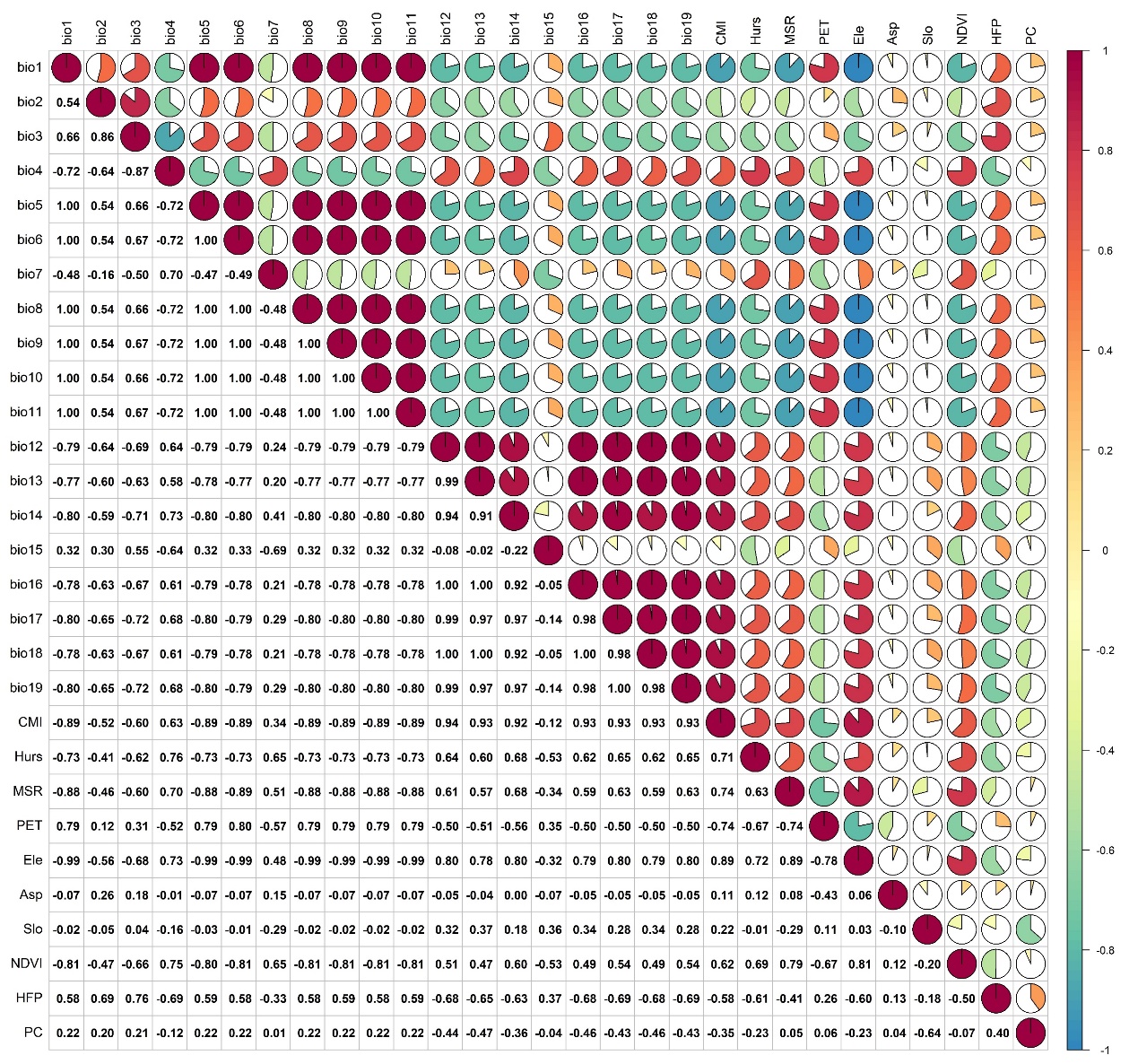
**

**Figure S7** Correlation matrix of environmental variables.

**Table S2** Results of multiple liner regression, variable importance of generalized boosted regression models (GBM) for Geographical Habitat Suitability Index (GHSI) for high suitable habitats relative to explanatory variables.

| **Variables** | **Estimate** | **SE** | **P** | **GBM** |
| --- | --- | --- | --- | --- |
| (Intercept) | 0.780 | 0.002 | < 0.001 | / |
| Asp | -0.002 | 0.003 | 0.473 | 3.38% |
| bio2 | -0.010 | 0.004 | 0.009 | 0.00% |
| bio4 | 0.011 | 0.007 | 0.133 | 8.13% |
| bio7 | 0.007 | 0.005 | 0.116 | 3.39% |
| bio12 | 0.024 | 0.006 | < 0.001 | 7.95% |
| bio15 | 0.013 | 0.005 | 0.007 | 1.61% |
| Hurs | 0.020 | 0.005 | < 0.001 | 3.55% |
| MSR | -0.010 | 0.005 | 0.023 | 16.66% |
| PET | -0.019 | 0.006 | 0.001 | 9.19% |
| Asp | -0.002 | 0.003 | 0.473 | 3.38% |
| Slo | -0.018 | 0.003 | < 0.001 | 10.86% |
| NDVI | -0.019 | 0.004 | < 0.001 | 16.32% |
| HFP | 0.058 | 0.004 | < 0.001 | 7.23% |
| PC | 0.005 | 0.002 | 0.061 | 11.74% |

**Table S3** The centroid ot the standard deviational ellipse (SDE) under projected current and future scenarios. XStdDist and YStdDist indicated the standard distance along longitude and latitude.

| **Centroid** | **Longitude** | **Latitude** | **XStdDist** | **YStdDist** | **Rotation** |
| --- | --- | --- | --- | --- | --- |
| current | 103.340376 | 29.54729 | 0.072294 | 0.048131 | 73.54032 |
| 2055 under SSP126 | 103.339425 | 29.54614 | 0.072389 | 0.048269 | 73.78296 |
| 2055 under SSP585 | 103.340873 | 29.54627 | 0.072356 | 0.048107 | 73.92498 |
| 2085 under SSP126 | 103.341212 | 29.54699 | 0.072182 | 0.048196 | 74679766 |
| 2085 under SSP585 | 103.338726 | 29.54552 | 0.071977 | 0.048353 | 73.2399 |
